# Supplementary material for: Quantifying archaeo-organic degradation – A multiproxy approach to understand the accelerated deterioration of the ancient organic cultural heritage at the Swedish Mesolithic site Ageröd
Source: PLoS One. 2020 Sep 23;15(9):e0239588. doi: 10.1371/journal.pone.0239588 (PMC7511028; doi:10.1371/journal.pone.0239588)
Supplement: Attachment — Submitted filename: Response to Reviewers.docx [file pone.0239588.s001.docx]

Reviewer #1: General comments: At least one author seems to have real trouble distinguishing between singular and plural. A lot of references to own publications rather than others which might be more relevant. All references to Boethius et al. (accepted) should be changed to Boethius et al. in print (unless that is a specific PLOS ONE way of referring to accepted manuscripts). It is published now so the full reference is now given.
-> means 'change to'.

Most are minor language or grammatic corrections.
Swedish 'kulturlager' translates into 'archaeological layer' or 'archaeological deposit', NOT 'cultural layer'! While it may not be the proper English term to use the word “cultural” we have chosen to do so to enhance comparability with the old excavation reports and the old publications concerning the 1940s and 1970s excavations at Ageröd, as this layer is there referred to either the “white layer” or the “cultural layer” or sometimes both, depending on the publication and context. Furthermore, to add with the comparability with our first Ageröd paper (Human encroachment…) we will write 'white (archaeological) cultural layer' the first time it is mentioned, after which we will write 'white cultural layer'
l.15: sites, Fixed
l.16: preservation, Fixed
l.23: have -> has Fixed
l.24: ..pHvalues in the still wet areas of the site have.. (delete two commas) Fixed
l. 26 oxidizes Fixed
l.31: ...within a decade through... Fixed
l.37, l.50, l.60, l.72, l.78, l.89, l.102, l.110, l.139, l. 373, l.386, l453, l.461, l.494, l.547, l. 563, l.585,l.592, etc.: accepted -> in print (systematic change of all following references to this text) Fixed
l.42: recovered on -> recovered at Fixed
l.45: perquisites -> prerequisites Fixed
l.63: made, Fixed
l.53: are -> is Fixed
l.65: was -> were Fixed
l.83: site, Fixed
l.90: available upon reques to the museum -> available at the museum upon request Fixed
l.93: decision -> decisions Fixed
ll.96-133: curious lack of any references to geoarchaeology and geochemical analyses outside Swedish borders and why these methods are preferred over others. References and comments added
l.98: samples -> sample
l.99: trench 201 and 205-> trenches 201 and 205 Fixed
l.101: from trench -> from trenches Fixed
l.105: delete cultural see above
l.107: cultural -> archaeological see above
l.108: layers, Fixed
l.113: what -> which Fixed
l.114: remains, Fixed
Table 1, Cit-P: delete double reference to Arrhenius, 1934 Fixed
l.169: from 1970s -> from the 1970s Fixed
l.176: following -> ensuing (because following is used in next line in same sentence) Fixed
ll.188-189: with- wash over -> with wash-over Fixed
l.189: Tolar et al., 2009 Fixed
l.193: fraction -> fractions; was -> were; partly -> only partly/ only in part Fixed
l.203: who -> which Fixed
l.206: Result -> Results (again in this chapter, a curious lack of any references to geoarchaeology and geochemical analyses outside Swedish borders and why the used methods are preferred over others used in the UK, DK, NL, NO...) References added see also similar comment above
l.216: delete culturalWhile it may not be the proper English term to also add the word “cultural” we have chosen to do so to enhance comparability with the old excavation reports and the old publications concerning the 1940s and 1970s excavations at Ageröd, as this layer is there referred to either the “white layer” or the “cultural layer” or sometimes both, depending on the publication and context
l.218: delete cultural See above answer

l.244: iron, Fixed
l.251: is -> being Fixed
l.255: trench -> trenches Fixed
l.256: which is -> something which is Fixed
l.257: slope, Fixed
l.258: reducing -> degrading (otherwise doesn't make sense when it is stated that the soil has completely oxidised - can't be reducing and oxidised at the same time); condition -> conditions Fixed
l.268: it -> there; active process -> active degradation process Fixed
l.275: relates -> relate Fixed
l.290: sample -> samples; who -> which Fixed
l.291: axle -> axis Fixed
l.303: lengthening (and -> lenthening, and; has -> have Fixed
l.307: delete cultural See above answer
Table 5: Wildboar -> Wild boar (3 occurences); good that Alces alces is stated as Elk/Moose cf. l. 363 Fixed
l.336: (F), Fixed
l.354: like a budding -> like budding Fixed
l.360: appear -> appears Fixed
l.361: delete cultural See above answer
l.362: layer, Fixed
l.370: analysed, Fixed
l.379: redox (good, should be used consistently in text - please change all uses of red-ox to redox) Fixed
l.384: show -> shows Fixed
Table 6: Wildboar -> Wild boar; Elk -> Elk/Moose (cf Table 5 and l.363 (5 occurences)) Fixed
l.395: suggest -> suggests Fixed
Fig 11 caption, ll. 399-402: please explain the difference between WL and CL??? The term 'archaeological cultural layer' is incorrect, delete cultural It is a mistake, thank you for noticing, WL and CL are the same WL has been deleted. See above answer
l.403: campaigns, Fixed
l.436: good; -> good, Fixed
l.439: cultural -> archaeological See above answer
l.465: done -> performed Fixed
l.477: reducing??? All following text indicates oxidising conditions. Please check! Reducing removed
ll. 477, 481, 489: Furthermore (used thrice, consider variation) Fixed
l.495: suggest -> suggests; are -> is Fixed
l.497: water level high stands -> high water level stands Fixed
l.500: occurs, Fixed
l.503: have -> has Fixed
l.510: as -> when; oxidate -> oxidates Fixed
l.517: delete cultural. See above answer

l.518: 2, Fixed
l.523: conditions, Fixed
l.527: its -> their Fixed
l.530: differential -> different; condition -> conditions Fixed
l.560: excavation -> excavations Fixed
l.563: suggest -> suggests Fixed
l.573: wheater -> weather Fixed
l.583: excavation -> excavations Fixed
l.593: add reference: McGovern, 2018 Done
l.596: who -> which Fixed
l.597: have -> has Fixed
l. 619: shown -> indicated Fixed

Reviewer #2: This paper presents an interesting and comprehensive analysis, which I think would be of interest to readers of PLOS one. The study is of great importance and highlights serious concerns about the future of wetland heritage. I enjoyed reading about such a fascinating site, and it is so unfortunate that its future is uncertain.
General points
Style and organization: I think that some improvement could be made in terms of the writing; please note that I have been picky in this regard because it was generally a rather pleasant read. In places however, the text is overly wordy and repetitive. I have pointed out some examples, but I do feel that the manuscript could do with another read over and some text removed to improve its readability. This is a particular problem in the “Discussion” section, where I feel that phrases such as “furthermore” and “in this context” have been added too often when sentences don’t actually link, and this has made it quite confusing to read. Removed the phrase “in this context” and removed the redundant “furthermores”

There is also some inconsistency in the writing style, particularly use of tenses (e.g. “we did” is used more in “collagen preservation” than other sections - this section doesn’t seem to match the rest of the manuscript). I don’t personally have a preference for passive tense as long as it is consistent but please refer to journal style. Fixed

A lot of the background information included in the “results” should really be in the “Discussion” section, and indeed in many places information is repeated in both. Please check for repetition and that information is in the appropriate place. We have remove the unnecessary repetitions

Introduction: In my opinion this is too brief. I would like to see more information of the other recent study at the site and how it relates to this one. I think the structure would be better if the site description was integrated here so that we already know its importance and history of excavation – it would make it easier to understand the context of this present study. Added: new sections to the introduction.

Some of the information in the “site description” section would be better in “Materials and Methods”. Expanded the site description somewhat and moved a section into Materials and Methods where we created a new section called “The excavation”.

I would also prefer a brief discussion of the analytical techniques used in the introductory section as they are an important part of the paper. Added: By investigating the soil chemical properties and by relating them to bone histological analyses, collagen preservation and the palaeobotany at the site, questions of how organic preservation has changed during the last seven decades and what might have caused the changes are answered and discussed.

On occasion I think there has been over interpretation of results and would like to see this resolved before publication. Amended at appropriate places following the specific points below

Finally, I think the title should be reconsidered. Archaeo-organic is a rather niche term that might not be very searchable, and I think the site name should be in the main title. In fact, I suggest you consider using your subtitle as the main title of the article: “A multi-proxy approach to understanding the accelerated deterioration of the ancient organic cultural heritage at the Swedish Mesolithic site Ageröd” To keep this manuscripts connection with our first paper about Ageröd, we think that the title should not change. However, we do think the suggestions is valid and since PLOS allows also a short title, we will change the short title to “A multi-proxy approach to understanding the accelerated deterioration at the Mesolithic site Ageröd” while keeping the whole title intact as it is.

Specific points
Line 23: “have already lost” should be “has already lost” Fixed
Line 26: “oxidize” should be “oxidizes” Fixed
Line 34: Please be consistent with the name of the site. Here you call it Ageröd I:HC but earlier it is just Ageröd, or Ageröd I. Are these different parts of the site? If so, please define what the I and/or HC means. Added: (section I:HC)
Line 38: there is a word missing here, perhaps “..degradation and revealed that in some areas….” Fixed
Line 39: What is meant by “hidden” here? It meant buried but we removed it for clarity

Line 45: “Prerequisites”? Fixed
Line 65: “which was used” should be “which were used” Fixed
Line 76: Do you really mean to say “arbitrarily”? Changed sentence to: The zone divisions were set to enable the study of differences in bone preservation…
Lines 92 and 96: Were there specific sampling criteria? Were the bone and soil samples selected at random? Yes, they were arbitrary selected where we could get a good soil sample and where we originally thought we could get good information. Had we done the sampling today, we would have included some more areas.
Line 98: “samples” should read “sample”. Fixed This description of samples is also quite confusing, perhaps reference to one of the tables would help. Added: (see Results section Tables 3 and 4)

Lines 113-115: here is an example of an overly wordy sentence (see general comment). Fixed I also do not know what is meant by “A variety of spectroscopic techniques of X-ray fluorescence….” As I don’t believe wet chemical, pH or MS are spectroscopic techniques? No they are not. It should be a comma instead of and, now fixed.

Table 1: I really like this, it’s very helpful. However, for Cit-P and Cit-POI you have explained the units of measurements in the “Method” column, whereas for previous methods it is in the “description” column. fixed
Lines 126-129: An example of repetition, this information is all in the Table. Removed repetition

Can information for XRF be put into the table? DOne

Line 135: I appreciate that that when dealing with so many different sample types it is confusing to explain, but in places it is not clear where samples are from or which are being discussed. Better reference to the tables could help with this. Added: (see Results section Table 5)

There is also some repetition which doesn’t help, you have already mentioned the selection of six samples from the previous excavations, I assume these are the same six – why not just mention them here for the first time? They are not the same and needs to be mentioned separately (though with better reference to the tables)

Line 141: The description of features would really be helped with some images. I know these do appear in Figures 7-9 – might it be better to discuss them in this level of detail in the results section so that the figures are there? Added : (see Results section for detailed images)

I also don’t think you have adequately explained the difference between OHI and GHI. Putting them together in Table 2 has made this even more confusing. Added explanation and an extra figure

Line 168: It is unclear whether these are the same bones used for histology. Some of them are, but most are not. This information is in Tab. 6.
Line 188: It would be clearer to say that 0.1 litre per sample was analysed (if this is correct!) Fixed
Line 193: “whereas 1-0.4…. these samples” is unclear. What does (ca1/10) mean? Why does the high organic content matter? Fixed by adding explanation

Line 196: *, ** and *** - what do they mean? Perhaps they refer to a figure; if so, the reference is missing. Yes they do, but we have the information in that figure so it is now removed from here
Line 206: “Result” should read “Results” Fixed
Line 208-209: An example of repetition / superfluous wording. This sentence is not necessary. It is necessary to name the table numbers. It does however not need to start the result section and we moved it to the end of the paragraph and shortened it somewhat.

Line 213: The meaning of “the potential corrosiveness of the sediment-bone relationship” is unclear. The large span does not necessarily indicate that the sediment is corrosive, rather the lowest pH value does. Changed to: This is a large span in the sediment pH, considering the limited size of the sampled area, and indicates highly dynamic sediment chemistry that in the lower pH range result in a rapid deterioration of the deposited bone materials.
Table 4: For readers who are unfamiliar with XRF data, I think you should remind us what the units are here. Added units to Table 4
Line 228: What does “different prerequisites” mean here? Very ambiguous, do you mean different soil chemistries? Added: (timing of formation, wetness, original peat thickness, subjection to groundwater fluctuation etc.)

Figure 4: I think this is a nice presentation of data but somewhat confusing. It could be improved by making it clearer that the colour refers to the trench number Added in figure text and putting the range of values onto each graph rather than in the legend. We tried but it looked worse, with too much information, so we think it better to keep the ranges within the figure text. The legend “depth variation correlated for pH and different elements” is ambiguous – I think something like “Variations in soil chemistry as a function of pH and depth” is a more accurate description. Fixed I also do not agree with the use of the term “element” here as organic matter is not an element Changed to component, and a lot of the description in the text underneath the legend should be in the legend itself, and does not need repeating in the text. The figure text will get really long if we put the text in here and we think it is easier to read if put in the main text and not in a figure legend, which is why we suggest keeping the text where it is.

Line 239: “shows pH as a function of…” is not true. The use of “as a function of” throughout this paragraph needs to be reconsidered. The figure shows pH as a function of depth, the magnitude of the soil chemistry parameters are plotted independently of these. Fixed as suggested
Line 264-265: It is unclear what the connection is between S content and “an initial and rapid decay of the OM”. Added, as the sulphur is not yet leached
Line 271 (para beginning): Fixed

I feel that the PCA has not been adequately described or justified. I actually do not see what it adds to the analysis and suggest either clarifying this or removing it entirely – you seem to conclude that pyrite causes differences in XRF data but that is obvious anyway. Many of the other conclusions / observations can be discerned from Figure 4 anyway, and to someone unfamiliar with PCA, there is no obvious separation of data points in either Figure 5 or 6. If it is left in, it needs far more explanation (e.g. p2t2 to p3t3? What does negative orientation mean? How does it relate to organic deterioration?) Added further explanation and a reference which explains the use of these methods in a PCA at length

Line 273: The use of phosphate content for “assessing general cultural impact” needs explaining. Changed to: The phosphate content is used for assessing the human impact on the archaeological deposit, as the phosphate content to a certain degree follow the impact and intensity during the formation of the layers.
Table 5: The weathering analysis needs explanation (i.e. which is worse, 1 or 5, what to the numbers in brackets mean?) The recently published Boethius et al. paper, referred to in the sentence, deals with these weathering analyses and to get a full understanding of the system used, the reader needs to read that paper as well. However, to facilitate reading this paper we added a small explanation here as well, as follows: (severity of degradation increases with increasing weathering numbers)

Line 311: A figure showing birefringence would be good (but I know it’s hard to show in a still). Figure added
Line 320-322: An example of an overly repetitive sentence. There are a few like this throughout and I do feel that it would benefit from a re-read. Rephrased to: In eight of the bones, some of these inclusions could be identified as framboidal pyrite, which are spherical clusters of smaller of iron sulphide (FeS_2_) crystals (Fig. 8)

Line 369: Is there a reason more bones weren’t studied? Yes, perhaps not the best reason, but nevertheless… We have, unfortunately, been deeply under financed for the project, with a lot of the research going on after normal working hours. We could not afford any additional analyses or thin section preparations, which is the main reason for not doing more analyses. Also, our permission agreement with Lund Historical Museum had a fixed number of destructive analyses specified, though we would likely have gotten a new permission to sample more bones if we had applied for it, as it currently stands we have used the maximal number of samples included in our permission.
Line 372: “deviant pattern… limited” very unclear what is meant by this, and how this relates to the weathering analysis. Yes, bad phrasing. Sentence changed to: Such bones as A6 would then not have come to define the histology of a whole zone, and the observations gained from singular bones would be limited, i.e. as illustrated by the weathering analyses where 3444 bones from the three excavation campaigns were used to quantify the status of the osseous remains (Boethius et al., accepted).

Table 6 contains a lot of information and I wonder if all of the columns are really necessary, particularly masses of sample and amount of collagen in mg as really it’s only the % that’s important to the interpretation. It is mainly about transparency of the data. We have not used any supplementary to this paper, choosing instead to present everything within the main text. Some of the data in this table could have gone into a sup. But since it would have been all that we would have put there we consider it more appropriate to put the information here and let the reader choose what he/she finds important.

Lines 389-393: Is all of this information really necessary? Shortened to: Because we were also interested in a chronological stratigraphic sequence, trench 201 was deemed best suited for these combined purposes; whereby, all bone samples used to test collagen preservation were selected from this trench
Line 403 (para beginning): This is very repetitive – most of this is in the figure legend as well. Changed to: To understand ongoing changes in collagen preservation, boxplots of the collagen yield from each excavation campaign were created.

Figure 12: I do not agree that this data is enough to establish a downwards trend and think that this has been over interpreted. There are not many data points for later excavations, and correlation is quite weak across only three time points. The prediction that the site will be lost by 2040 assumes deterioration is linear, and you can’t know that from this study. It can perhaps be presented as a hypothesis, but I have a problem with the way this has been presented here as fact. Added: hypothetical and might and

Table 7: legend says 6 samples, it should be 7 Fixed
Figure 13: Do you really mean “collation” - maybe “selection”? Yes, fixed
Line 447: The link between the presence of corrosion damage and the material being of Mesolithic origin needs clarifying. Can corrosion not have been caused at any time period? Yes, changed to: Corrosion damage was observed on both water pepper seeds (Fig. 17a) and hazelnut shells (Fig. 17b) from trench 209. Currently it is not known when this corrosion occurred, but it is conceivable that it coincide with the bone deteriorating processes.

Line 466: “Indications” should be “indicators” Fixed
Line 458: In general, I find this discussion repetitive from the previous sections and the whole thing could do with editing, with some sentences shortened or deleted. We have gone through the text and shortened it somewhat.
Line 468: The decomposition of peat as determined by what parameter – can you refer to a table? Do you maybe mean that the soil chemistry varies? Added following:, as determined by the soil chemical properties (Tables 3 & 4),

Line 473: I think you need to acknowledge that you may not have found bones in these trenches simply because there never were bones. I don’t think it takes from the study at all, because there clearly IS significant deterioration, but there are limitations on the data – your data set is (understandably) very small. Added following: Assessments of the bone deposition pattern of the site, related to rectified GIS-data, enabled the positioning of the 2019 test trenches in the direct vicinity of where the best preserved and most numerous bone remains had been recovered on the previous excavations of the site [1], which suggest that it is unlikely that there would have been no bone remains originally deposited in the 2019 trenches.

Line 481: Again I think there has been some over interpretation here. There is only one sample from trench 209, so can you really say it is “characteristic”? How do you know that the difference isn’t due to the groundwater level? I think perhaps this point has not been discussed very clearly. Yes, characteristic is the wrong term, it is now changed to “are demonstrated” We do not say that it is not connected to the groundwater we state that it “implies that the much lower pH in trench 209 compared to 205 is connected to the groundwater”

Line 487: decimeters is quite an unusual term to use, and for the first time in this manuscript. Changed to meters
Line 499: You cannot know that bones have been dissolved – what evidence is there that they ever existed? Our test trencher were carefully selected and located in the direct connection to the square metres where the largest amounts of bones had been recovered in the 1940s or the 1970s. Thus while cannot “know” with 100% certainty, we have taken all measures to ensure that the trenches we excavated did indeed have the prerequisites to have originally held lots of bones. Added further explanation to the introduction

The phrase “But remember that…” is a bit odd in the tone of the paper. Changed to: However

Line 506: “suggests that there is a contribution of iron to the soil” is superfluous. Please delete. Done
Line 516: “completely bioeroded” implies that it has disappeared! Removed “completely”

Line 524: Are you sure that this means conditions have been stable until the 1940s/70s? Can pyrite not have formed at any time between deposition and excavation? Yes, the pyrite can have formed at any point, but is has not oxidated until after 1970s (at least as we write at parts of the site), which suggest that the conditions were fairly stable, see also answer to next question below.

Line 565: I am not convinced from the discussion here that it has actually been proven that pyrite oxidation occurred so recently. Perhaps this needs communicating a little better? We only have intact pyrite grains from the old excavations and we only have oxidated pyrite grains from our new excavation, which is why we concluded that the pyrite have just recently oxidated. Added further explanation: (which, as shown through the recovery of only intact pyrite grains within the bones from the old excavations while all of the detected pyrite grains in the 2019 bones had oxidized, did not start until after the 1970s)

Line 573: “wheater” should be “weather” Fixed
Line 575: Check reference. Yes, it is a strange reference although it does look like this, there is no author stated as it is given according to the suggested citation as presented on the inside of the book cover.
Line 576: “Complete destruction of the bone remains” – please reconsider this hypothesis! Added additional text to sentence: (though further investigations are needed to understand if the groundwater have turned acidic and dissolved the osseous remains throughout the bog or if these effects are limited to the vicinity of the old excavations)
Line 597: “have declined” should read “has declined” Fixed

Line 602 (para beginning): These are very important conclusions, and passionately communicated. May I suggest that you might want to highlight the fact that changing climates will make it more and more difficult to maintain stable conditions at sites like this? Do you have suggestions on what can be done – do you propose simply to excavate? I also suggest that you repeat some of this urgency and passion in the abstract of the manuscript, in case it is all that someone reads. Added a sentence with suggestion of how problems can be “fixed”: Thus, to enable future organic preservation, both at Ageröd and at similar sites elsewhere, pH levels in both soil and water must first be made neutral before groundwater levels can be raised and actions to recreate anaerobic burial conditions can be initiated. And added a bit of “passion” to the abstract
